# Consumption patterns of legumes and related dietary guidelines across Europe: a specific focus on peas and chickpeas

**DOI:** 10.3389/fnut.2026.1770686

**Published:** 2026-03-26

**Authors:** Carmen Grosch, Kurt Gedrich

**Affiliations:** Technische Universität München, ZIEL – Institute for Food & Health, Freising, Germany

**Keywords:** food consumption, food-based dietary guidelines, Europe, legumes, pulses, peas, chickpeas

## Abstract

Legumes such as peas and chickpeas hold a variety of beneficial qualities. Therefore, their consumption is recommended by several international bodies. To understand how different countries in the European Union judge the importance of legumes as a part of a healthy and sustainable diet, we compared the current food-based dietary guidelines (FBDG) in the EU member states. In most dietary guidelines across Europe, legumes are categorized either as “fruit and vegetables” or as “proteins.” Only the Portuguese guidelines define legumes as a separate food category (i.e., pulses). In seven countries, legumes were suggested as a suitable substitute for animal proteins. Spain and Portugal had the highest recommendations (at least 4 portions a week and 1–2 portions per day, respectively). Serving sizes varied greatly from country to country, ranging from 60 g in the Netherlands to up to 300 g in Bulgaria (cooked weights). To estimate recommended annual consumption, both serving sizes and recommended frequencies were considered for countries that provided respective information. Spain and Portugal emerged with the highest amounts (approx. 30–60 kg/year). In contrast, the lowest recommendations are found in the Netherlands and Germany (approx. 6 kg/year). A cross-country comparison reveals notable variation in FBDG, even among culturally similar countries like Austria and Germany. It shows that unambitious legume recommendations in FBDG reflect a cultural unfamiliarity that persists even as environmental and health imperatives grow.

## 1 Introduction

The global food system is increasingly burdened by challenges such as climate change, resource depletion, biodiversity loss, and persistent food insecurity (1). To address these issues, the concept of sustainable food systems has gained increasing attention. According to the Food and Agriculture Organization of the United Nations (2), a sustainable food system ensures food security and nutrition for current and future generations. Realizing such a system requires a careful balance across three core dimensions, i.e., environmental, social, and economic sustainability, to establish a resilient, inclusive, and resource-efficient food network (3).

The transition toward a sustainable food system relies on diversifying protein sources and reducing agriculture-related emissions (2). In this context, underutilized crops, also referred to as orphan or neglected species, are receiving renewed interest. These crops were once widely cultivated and consumed but have declined due to various agronomic, economic, and social factors (4). They are typically highly nutritious, culturally significant, and well-adapted to marginal environments. Their wider acceptance into modern food systems is usually hindered by limited competitiveness, exclusion from policy and research agendas, and loss of traditional knowledge (4).

The classification of crops as underutilized is based on regional context: a species may function as a dietary staple in one region while remaining underrepresented in another, even where growing conditions are favorable (4). Legumes, particularly peas and chickpeas, are prime examples of such underutilized crops in the European context. While they have historically played an important role in traditional diets and crop rotations, their cultivation has declined substantially in recent decades (5). According to the German Bundesanstalt für Landwirtschaft und Ernährung (BLE), for instance, their cultivation covered just 2.2% of Germany’s arable land in 2024 (6).

Despite their marginalized status, legumes offer a compelling solution to contemporary agricultural and nutritional challenges. Prioritizing legumes like peas and chickpeas promises various benefits: not only do they enhance soil health through nitrogen fixation, but they also provide nutrient-dense, plant-based protein. As low-input, climate-resilient crops, they represent a strategic entry point for making European agriculture more sustainable (7).

Food systems shape not only what we eat, but also how we live together. A truly sustainable food system must therefore go beyond ecological and economic concerns to address social and cultural dimensions of sustainability. This includes ensuring equitable access to adequate nutrition and fostering cultural inclusivity, particularly for marginalized and vulnerable population groups (2). In line with this, the Food and Agriculture Organization (FAO) and the World Health Organization (WHO) jointly define a sustainable healthy diet as dietary patterns that are nutritionally adequate, culturally acceptable, economically fair, and environmentally responsible (8).

Legumes such as chickpeas and peas hold considerable potential in advancing the social sustainability of food systems. Cultivated for millennia, they are deeply ingrained in many traditional cuisines around the world, appreciated both for their nutritional value and culinary versatility (7). Reintegrating these underutilized crops into modern food systems may therefore help bridge the gap between nutrition, sustainability, and cultural relevance.

In light of the numerous beneficial qualities of legumes (9), their consumption is recommended by several international bodies. To understand how different countries in the European Union assess the importance of legumes as part of a healthy and sustainable diet, we compared the current Food-Based Dietary Guidelines (FBDG) in EU member states with a specific focus on the representation of legumes. In doing so, we aim to identify challenges and opportunities for reintegrating legumes into both agricultural practices and dietary patterns, contributing to the broader goal of more sustainable and resilient food systems.

## 2 Method

To examine the standing of legumes in dietary guidance, a comparative review of FBDG across all the 27 EU Member States was conducted. These were sourced from FAO’s online-repository of FBDG (10). If information on those FBDG was not provided in English, DeepL was used for translations.^1^

Each FBDG was assessed to determine whether legumes were classified as a source of protein, as part of the “fruits and vegetables” group, or regarded as their own food category. Where specific serving sizes were provided, these were extracted. If no quantitative recommendation was stated, data were marked as “Not Available” (N.A.).

Guidelines were also analyzed for explicit recommendations of legumes as a replacement for animal-based products. Only those documents that clearly advised substituting animal products with legumes were considered, i.e., general references to legumes as an advisable source of plant-based protein were not regarded as sufficient.

To facilitate comparison, the information in the FBDG was converted into recommended annual legume consumption (in kilograms per person) if recommended consumption frequencies as well as serving size data were provided. Wherever feasible, cooked quantities were used to improve cross-national comparability. Mean values of recommended annual legume consumption were calculated by averaging the lower and upper quantities across EU-27 countries with available data. Where only one value was provided, it was used for both the minimum and maximum quantities.

## 3 Results

Table 1 summarizes the classification and consumption recommendations for legumes in the national FBDG across EU Member States. In most guidelines, legumes are categorized either as part of the fruit and vegetable group (9 out of 27) or as a source of protein (17 out of 27). Only the Portuguese guidelines define legumes (pulses) as a separate food category.

**Table 1:** Classification and consumption recommendations for legumes in national FBDG.

Country	Classification	Recommended frequency (servings/week)	Recommended Serving Size	
			Raw/Dry	Cooked
Austria	F&V	3–4		125 g
Belgium	Protein	1+		N.A.
Bulgaria	Protein	2+		200–300 g
Croatia	Protein	N.A.		N.A.
Cyprus	F&V	2–3+		1/2 cup (90 g)
Czechia	Protein	1		N.A.
Denmark	Protein	7		100 g
Estonia	F&V	3–4	30 g	90 g^*^
Finland	F&V	More		100 ml
France	F&V	2+		N.A.
Germany	F&V	1	70 g	125 g
Greece	Protein	1+		150–200 g
Hungary	F&V	1+		N.A.
Ireland	Protein	N.A.		3/4 cup
Italy	Protein	2–4	50 g	150 g
Latvia	Protein	More		1 glass
Lithuania	Protein	2–3		N.A.
Luxembourg	Protein	N.A.	40–50 g	150–220 g
Malta	Protein	2+	70 g	140 g
Netherlands	Protein	2–3		60g
Poland	Protein	More		N.A.
Portugal	Pulses	7–14	25 g	80 g
Romania	F&V	N.A.		1/2 cup
Slovakia	Protein	More		N.A.
Slovenia	Protein	N.A.		N.A.
Spain	Protein	4–7	50–60 g	170 g
Sweden	F&V	More		N.A.

* Cooked serving size is estimated based on an average conversion factor of 3.0.

Abbreviations: Protein: Source of Protein; F&V: Part of fruit and vegetables; N.A.: Not available.

Spain and Portugal had the highest recommendations (at least 4 portions a week and 1–2 portions per day, respectively). The underlying serving sizes varied substantially from country to country and were given as either dry or cooked weights. Based on raw or dried weights, the suggested serving sizes range from 25 g in Portugal to 70 g in Germany or Malta, whereas the cooked serving sizes range from 60 g in the Netherlands to up to 300 g in Bulgaria.

Five countries (Poland, Slovakia, Finland, Latvia, and Sweden) recommended increasing legume intake, without specifying the advised frequency of consumption. Another five countries specified the recommended frequencies but did not define respective serving sizes (Belgium, Lithuania, France, Czechia, and Hungary). Finally, two countries, Croatia and Slovenia, specified neither recommended frequencies nor serving sizes.

In 12 countries, the FBDG comprised recommended frequencies as well as serving sizes, allowing for the calculation of recommended consumption quantities per year (c.f. Table 2). Again, Spain and Portugal emerged with the highest amounts. In Portugal, recommended annual legume intakes range from 29.1 kg to 58.2 kg (based on either one or two portions of 80 g cooked weight per day). In Spain, the recommendation ranges from 35.4 to 61.9 kg of cooked weight per year. In contrast, the lowest recommendations are found in Germany (6.5 kg/year cooked weight) and the Netherlands (6.2–9.4 kg/year cooked weight). Among those EU member states that provide specific recommendations on legume intake (including frequencies and serving size), the average recommended annual consumption ranges from 18.5 to 26.5 kg/year of cooked legumes.

**Table 2:** Recommended annual legume consumption based on national FBDG across EU member states.

Country	Recommended cooked quantities (kg/year)		
Austria			26.0
Bulgaria	20.8	–	31.2
Cyprus	9.4	–	14.0
Denmark			36.4
Estonia	14.0	–	18.7
Germany			6.5
Greece	7.8	–	10.4
Italy	15.6	–	31.2
Malta			14.6
Netherlands	6.2	–	9.4
Portugal	29.1	–	58.2
Spain	35.4	–	61.9
Average	18.5	–	26.5

In seven countries, the FBDG explicitly suggest legumes as suitable substitutes for animal proteins. Those are Italy, the Netherlands, Spain, Belgium, Bulgaria, Denmark, and Poland (Table 3). In most cases, legumes are cited as examples of plant-based protein sources. Only the Belgian recommendations focus specifically on legumes, suggesting that meat be replaced at least once a week with legumes, which can be fresh, dried, frozen, canned, or pureed.

**Table 3:** Substitution messages for legumes in national Food-Based Dietary Guidelines (FBDG) across EU Member States.

Country	Substitution message
Belgium	Replace meat at least once a week with legumes. They can be fresh, dried, frozen, canned, or pureed.
Bulgaria	Replace meat and meat products often with fish, poultry or pulses.
Denmark	Introduce meat-free days and cut down on meat in your meals. You can replace meat with vegetables, legumes, or wholegrains.
Italy	Reduce red meat in favor of poultry or plant-based protein.
Netherlands	Eat less meat and more plant-based proteins like pulses and nuts.
Poland	For health and environmental benefits, replace meat with plant-based protein sources such as legumes (beans, chickpeas, soy, peas, lentils, broad beans) and nuts, as well as fish and eggs.
Spain	It is recommended to prioritize the consumption of plant-based foods over animal-based foods as the main sources of protein in the diet. The consumption of vegetable protein, mainly legumes, should occupy one of the protein rations of the main daily meals (food and dinner).

## 4 Discussion

### 4.1 Cultural resistance: why legumes struggle on the plate

Cross-country comparisons reveal notable variation in FBDG, even among culturally similar countries. For example, Austria recommends consuming at least 375 g of cooked legumes per week, whereas the German guidelines suggest only a third of that amount. These rather low German values are the result of a mathematical optimization approach. The German FBDG aim to reduce the burden of disease, minimize environmental impact, and maintain alignment with current eating habits. Regardless of the modelling scenarios, the recommended legume intake remained constant, i.e., one portion per week (11), which translates to approximately 6.5 kg/year of cooked legumes. While this represents a clearly defined and practical recommendation—unlike the vague “increase consumption” phrasing used in many other EU guidelines—it remains comparatively unambitious. However, the German recommendations also reflect a cultural unfamiliarity that persists, even as environmental and health imperatives intensify.

While production challenges are considerable, consumer habits and socio-cultural perceptions also pose significant obstacles to the broader integration of legumes into European diets. Understanding why cultural resistance persists is a crucial first step toward shifting long-term dietary habits.

Consumption patterns are shaped not only by product availability and price, but also by tradition and long-established preferences (12). In countries such as Italy, Spain, Greece, and Portugal, legumes are a well-established component of the Mediterranean Diet, which emphasizes plant-based foods including pulses (7). Nevertheless, legume consumption is declining even in these regions (13), highlighting the fragility of these traditions in modern food systems.

Behavioral studies further illustrate the resistance to dietary change. Based on a review of 38 studies, Hartmann and Siegrist (14) report that the willingness to reduce meat consumption and the readiness to adopt meat substitutes are both generally low. They also noted a widespread underestimation of the environmental impact of meat consumption. Henn et al. (15) observed a similar pattern in relation to pulse consumption: individuals unfamiliar with legumes as meat alternatives were unlikely to change their eating habits in favor of legumes. Their findings point to a clear socio-cultural divide, with higher levels of education and dietary identity (e.g., vegetarian, vegan, flexitarian) being strongly associated with more frequent consumption of pulses.

In addition to cultural resistance, practical barriers also deter the consumption of legumes. Many consumers perceive legumes as inconvenient due to time-intensive preparation and a lack of culinary familiarity (16). Notably, traditional concerns such as flatulence played only a minor role. These perceptions persist despite increasing awareness of their health and environmental benefits, underscoring the need for targeted interventions that address both cultural norms and everyday usability.

The need for such interventions becomes particularly evident when considering the currently low levels of legume consumption. In Germany, for example, per capita intake averages just 5 grams per week (17), a figure that falls significantly short of both national dietary recommendations and international benchmarks. Bridging this gap between recommended and actual intake is therefore an essential step toward fostering more sustainable and health-promoting dietary patterns.

### 4.2 From niche to norm: strategies for legume adoption

Despite growing awareness of their nutritional value and environmental benefits, legumes remain underutilized in many European diets (15). According to the most recent data in the Comprehensive European Food Consumption Database published by the European Food Safety Authority (18), the average consumption of legumes by European adults ranges from below 4 g/d in countries such as Austria, Belgium, Germany, the Netherlands, or Sweden up to about 12 g/d in countries such as Cyprus, Hungary, Italy, Portugal, or Serbia. Overall, this makes roughly just 15% of the reference diet suggested by the EAT-Lancet Commission (19). Therefore, addressing perceived inconvenience and enhancing consumer familiarity are central challenges and opportunities for sustainable food system transformation.

A key actor in this shift is the food industry, which plays a pivotal role in developing palatable and convenient legume-based products tailored to modern lifestyles. Innovations such as chickpea tempeh or the Brandenburger Kichererbsentopf, a regional chickpea stew, are examples of how legumes can be reimagined in ready-to-eat formats. Such innovations portray important strategies to address barriers, such as perceived inconvenience, that deter consumers from integrating legumes into their diets (16).

Equally important is the role of consumer education and institutional catering. Initiatives like LeguNet take a structural approach to normalizing legume consumption, not through traditional marketing in convenience stores, but by targeting communal settings such as schools, workplaces, and hospitals. These environments provide consistent exposure to legume-based meals and are particularly effective in reaching diverse population groups, including children, older adults, and employees who may not typically include legumes in their regular diets. Such settings not only shape long-term dietary habits but also support regional producers by generating reliable demand through bulk purchases and stable supply chains (20).

This approach might also be closely aligned with national policy goals. For instance, the German Federal Ministry of Agriculture, Food and Regional Identity (Bundesministerium für Landwirtschaft, Ernährung und Heimat, BMLEH) emphasizes that institutional catering should prioritize sustainable, affordable meals built around plant-based, seasonal, and regional foods. With approximately 16 million individuals relying on institutional food services in Germany alone (21), this sector represents a major leverage point for repositioning legumes.

### 4.3 Limitations

While dietary guidelines were analyzed, we did not include primary research on cultural attitudes toward legumes. Given that consumer acceptance is a critical factor influencing the practical relevance of legumes such as peas and chickpeas, future research should explore evolving consumption trends and assess the effectiveness of strategies aimed at increasing the visibility and appeal of legumes.

Furthermore, the agronomic potential of legumes is highly context-specific. The adaptability of chickpeas and peas to different European climate zones requires further investigation. We did not evaluate specific geographic regions where these crops may offer a viable alternative, particularly in the face of climate change. Field trials and region-specific studies are crucial for evaluating their practical suitability and long-term potential.

## 5 Conclusion

Fostering underutilized legumes such as peas or chickpeas into European food systems presents a tangible opportunity to address environmental, nutritional, and agronomic challenges. Despite their well-documented contributions to soil health, dietary diversity, and climate mitigation, peas and chickpeas remain underutilized—both in cultivation and consumption. While policy instruments such as national dietary guidelines increasingly acknowledge their value, they do not go far enough in removing structural and perceptual barriers to their broader integration. Thus, overall production and consumption levels remain limited.

Achieving genuinely sustainable food systems will require more than technical innovation. Legumes must be repositioned—from niche alternatives to essential components of agroecological and nutritional strategies. To enable this shift, ambitious and aligned action is needed across policy, practice, and perception. Reforming FBDG to align with EAT-Lancet targets (22) and clearly recommending legumes as central to sustainable and healthy diets could provide a clear incentive for increased legume consumption.

Likewise, increasing visibility, especially in schools and institutional catering, could help normalize legume-based meals and establish reliable demand (23). The underrepresentation of chickpeas and peas is not a reflection of their potential, but of the systems that shape their use (24). Initiatives such as vocational education programs and school-based outreach—like those implemented by LeguNet (20)—demonstrate how bottom-up approaches can help increase visibility, shift perceptions, and complement top-down policy reforms.

Ultimately, the future of peas and chickpeas lies not only in the fields, but at the center of the plate. The challenge is not simply to recognize their agronomic and nutritional value, but to restore their place within food culture—accessible, familiar, and desirable to the broader public. In doing so, they can fulfil their full potential as a pathway toward a more resilient, inclusive, and ecologically sound food system in a world with a rapidly changing climate.

## Footnotes

1
https://www.deepl.com/de/translator
